# Peripheral Nerve Injury Induced by Japanese Encephalitis Virus in C57BL/6 Mouse

**DOI:** 10.1128/jvi.01658-22

**Published:** 2023-04-18

**Authors:** Huan Yang, Xiaoli Wang, Zhao Wang, Guowei Wang, Shihong Fu, Fan Li, Liping Yang, Yanping Yuan, Kaichun Shen, Huanyu Wang, Zhenhai Wang

**Affiliations:** a General Hospital of Ningxia Medical University, Yinchuan, China; b The No. 1 People’s Hospital of Shizuishan, Shizuishan, China; c The Second Affiliated Hospital of Air Force Medical University, Xi'an, China; d Ningxia Medical University, Yinchuan, China; e Department of Viral Encephalitis, NHC Key Laboratory of Biosafety, National Institute for Viral Disease Control and Prevention, Chinese Center for Disease Control and Prevention, Beijing, China; f State Key Laboratory for Infectious Disease Prevention and Control, Chinese Center for Disease Control and Prevention, Beijing, China; g Institute of Medical Sciences, General Hospital of Ningxia Medical University, Yinchuan, China; h Diagnosis and Treatment Engineering Technology Research Center of Nervous System Diseases of Ningxia Hui Autonomous Region, Yinchuan, China; i Neurology Center, General Hospital of Ningxia Medical University, Yinchuan, China; University of North Carolina at Chapel Hill

**Keywords:** JEV, PNI, C57BL/6 mouse, sciatic nerves, myelin sheath, axon

## Abstract

Japanese encephalitis virus (JEV), with neurotoxic and neuroinvasive properties, is the major cause of human viral encephalitis in Asia. Although Guillain-Barré syndrome caused by JEV infections is not frequent, a few cases have been reported in recent years. To date, no existing animal model for JEV-induced peripheral nerve injury (PNI) has been established, and thus the pathogenic mechanism is not clarified. Therefore, an animal model is urgently required to clarify the correlation between JEV infection and PNI. In the present study, we used JEV GIb strain of NX1889 to establish a mouse model of JEV infection. The general neurological signs emerged on day 3 of modeling. The motor function continued to deteriorate, reaching a maximum at 8 to 13 days postinfection (dpi) and gradually recovered after 16 dpi. The injuries of 10^5^ PFU and 10^6^ PFU groups were the most severe. Transmission electron microscopy and immunofluorescence staining showed varying degrees of demyelination and axonal degeneration in the sciatic nerves. The electrophysiological recordings demonstrated the presence of demyelinating peripheral neuropathy with reduced nerve conduction velocity. The decreased amplitudes and the prolonged end latency revealed axonal-type motor neuropathy. Demyelination is predominant in the early stage, followed by axonal injury. The expression level of JEV-E protein and viral RNA was elevated in the injured sciatic nerves, suggesting that it may cause PNI at the early stage. Inflammatory cell infiltration and increased inflammatory cytokines indicated that neuroinflammation is involved in JEV-induced PNI.

**IMPORTANCE** JEV is a neurotropic flavivirus belonging to the *Flaviviridae* family and causes high mortality and disability rates. It invades the central nervous system and induces acute inflammatory injury and neuronal death. Thus, JEV infection is a major global public health concern. Previously, motor dysfunction was mainly attributed to central nervous system damage. Our knowledge regarding JEV-induced PNI is vague and neglected. Therefore, a laboratory animal model is essential. Herein, we showed that C57BL/6 mice can be used to study JEV-induced PNI through multiple approaches. We also demonstrated that viral loads might be positively correlated with lesion severity. Therefore, inflammation and direct virus infection may be the putative mechanisms underlying JEV-induced PNI. The results of this study laid the foundation for further elucidation of the pathogenesis mechanisms of PNI caused by JEV.

## INTRODUCTION

Japanese encephalitis (JE), caused by the Japanese encephalitis virus (JEV), is a potentially fatal, vector-borne viral disease. JEV is an enveloped virus with a single-stranded positive-sense RNA genome and a member of the *Flaviviridae* family (1–3). On a global scale, JE is considered the most common epidemic of encephalitis in humans and animals, widely endemic in Northern Australia, the Asian Pacific Rim, South and Southeast Asia, Eastern Russia, and the Western Pacific Region (4, 5). Approximately 68,000 cases of JE in humans occur in Asian countries annually, with a mortality rate of 20 to 30%; among them, 30 to 50% of survivors suffer permanent neurological, behavioral, and cognitive sequelae (6, 7). Individuals of any age can suffer from JE, although young children are at a higher risk (8). JEV has been divided into five distinct genotypes (GI to GV) based on the analysis of the envelope gene or complete genome sequences, with genotypes I and III as predominant (9, 10). Recently, GI strain has replaced GIII as the dominant genotype in most Asian countries (11). While the JE vaccine has a remarkable impact on preventing the spread of the disease, no approved efficient antiviral therapy directed against JEV is currently available (12).

Animal models can provide valuable information about virus pathogenesis, vaccine efficacy and safety, and antiviral drugs or therapeutics assessment. Animal models, such as mice, hamsters, guinea pigs, swine, rats, monkeys, and rabbits, have been employed for studying JEV pathogenesis (13). However, compared to other animals, mice constitute a more economical, well-established, and broadly used experimental model for understanding JEV neuropathogenesis (14, 15). The inoculation of JEV into the mouse model produces characteristic clinical signs (fever, loss of body weight, hair erection, hunched back, body stiffening, whole-body tremor, abnormal gait, seizures, restriction of movement, and paralysis of hind limb) and histopathological lesions of encephalitis (16). The clinical features of JE in humans are diverse, ranging from asymptomatic to severe neurological symptoms and death, including a nonspecific fever, vomiting, headache, signs of meningitis, irritation, recurrent seizures, speaking inability, focal neurologic deficits, a parkinsonian syndrome, a polio-like acute flaccid paralysis, and loss of consciousness (4, 17, 18).

C57BL/6 is the most extensively used inbred mouse strain in JEV studies. Owing to its flexible neurological signs and the preferential homing of JEV to the central nervous system (CNS), C57BL/6 mice are primary candidates for studying neurophysiology, neuroethology, and neuropathology of JEV infection (19). According to the association between infection efficiency and the growth process, 4- to 6-week-old mice are susceptible to JEV (20), and the interferon-deficient model with severe clinical manifestations is considered optimal for related studies (21). However, we cannot judge how much difference in interferon (IFN) response would effectuate the natural pathogenic mechanism of JEV. Considering that the pathogenicity of flaviviruses may be measured based on the effect of the virus and the resulting immune response, an immunocompetent mouse model is an urgent requirement to elucidate the pathogenesis of JEV.

The mechanisms of JE, including neuroinflammation (22), neuroinvasion (23), neuroimmunity, neurocytes damage or death (24), and mitophagy (25), are complicated and have not been elucidated; nonetheless, some progress has been made in recent years. The acute flaccid paralysis associated with JEV infection is caused by extensive brain lesions (26) and degeneration and death of motor neurons in the anterior horn of the spinal cord (27). The largest outbreak of JEV infection in 2018 occurred in Ningxia, China. The clinical features indicate a moderate to high degree of severity in addition to symptoms of CNS injury; also, the flaccid paralysis symptoms are pronounced, and the electromyography (EMG) results are consistent with Guillain-Barré syndrome (GBS) (28). Although peripheral nerve injury (PNI) induced by JEV has rarely been reported, Zika and dengue viruses of the same *Flaviviridae* family have already been widely reported (29–31). Hitherto, only a few studies have reported the pathogenesis of JEV-mediated PNI, partially due to the difficulty in performing clinical trials on humans during JEV outbreaks. Although the current mouse model cannot fully replicate the human clinical features, it mimics the classical JEV neurological signs and pathological changes in the brain tissue. Therefore, we aimed to establish a reliable mouse model to recapitulate the human pathogenesis of JEV-induced PNI. In the present study, varied doses of JEV were inoculated in the mice; then, the animals were observed continuously for the general clinical signs, motor function defects, and pathological changes to determine the application of the mouse model in JEV-induced PNI.

## RESULTS

### General characteristics of mice after JEV inoculation.

We inoculated mice with varying doses of JEV to elucidate the severity and time course of the defects. Mice were monitored daily for hair, weight loss, active state, and survival. The scores of disease severity or 20% loss of initial body weight were used as termination criteria. Mice in the sham group had a diet of water, normal behavior, and smooth and dense hair. Conversely, the hair of mice in viral groups was rough, loose, and poorly glossy (Fig. S1 in the supplemental material). Poor mental state (somnolence, slow response, less exploratory, and decreased appetite) and reduced activity were observed in virus-inoculated mice, and the 10^5^ PFU and 10^6^ PFU groups presented the most severe conditions. The general clinical signs appeared on day 3 of modeling. Most mice presented typical neurological signs at 4 to 8 days postinfection (dpi), manifested by hunch posture, tremors, lethargy, circling, ataxia, slow movements, abnormal gait, and hind limb paralysis. This phenomenon continued to deteriorate and reached a peak at 8 to 13 dpi (seizure-like activity, burnout, weakness, and paresis). The mice were euthanized in accordance with IACUC-approved criteria as they reached the standard clinical endpoints, while the remaining mice were observed and appeared to recover after 16 dpi.

**FIG 1:**
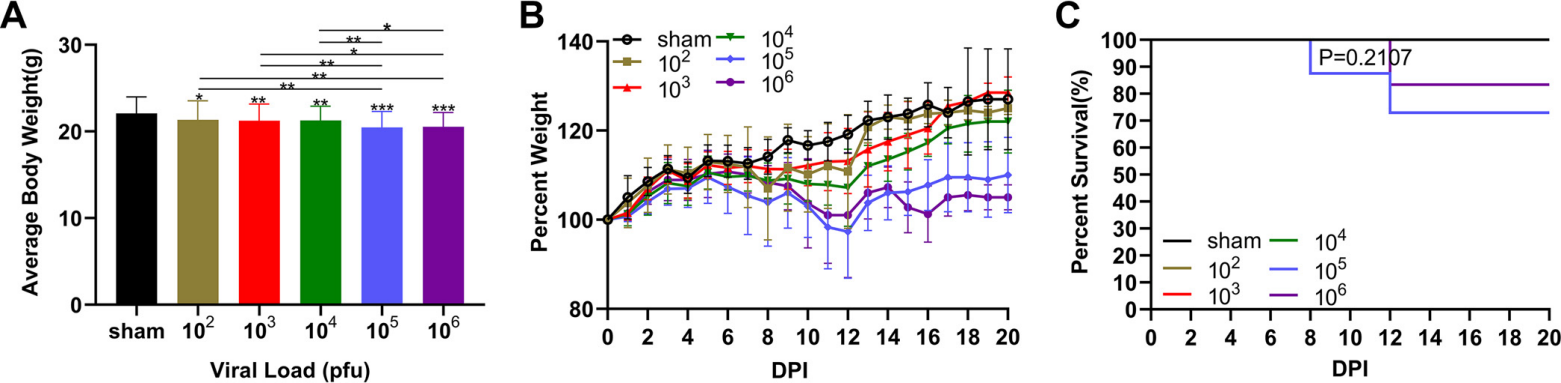
Weight loss and survival curves of C57BL/6 mice. (A) Absolute body weight of the different groups (*n* = 10/group). One-way ANOVA with Bonferroni’s multiple comparison test was used to compare differences among multiple groups. (B) Changes in body weight expressed as a percentage of starting weight for 20 days after the virus challenge. (C) Percentage of survival of each group surviving each day after JEV infection (*n* = 10/group). In the 10^5^ PFU group, one mouse died at 8 dpi and another died at 12 dpi. In the 10^6^ PFU group, only one mouse died on 12 dpi. Survival curves were compared using log-rank Mantel-Cox curve comparison. Data are expressed as means ± SD. *, *P* < 0.05; **, *P* < 0.01; ***, *P* < 0.001.

No significant differences were detected in the initial body weights among the different groups (*P* > 0.05). The analysis of the change in body weight depicted that the virus-challenged group mice suffered, while the sham mice did not suffer from significant growth retardation. Conversely, the 10^5^ PFU and 10^6^ PFU groups lost substantial body weight compared to that of the other groups. However, the weight changes were similar between the two groups (Fig. 1A). The weight of mice in the six groups increased rapidly for 0 to 3 dpi. In contrast, the mice in the different virus groups showed obvious differences after 5 dpi. The 10^2^ PFU, 10^3^ PFU, and 10^4^ PFU groups displayed a significant decreasing weight trend from 8 to 12 dpi and started to regain weight after 13 dpi. The 10^5^ PFU and 10^6^ PFU groups initially experienced a gradual decline in weight from 5 to 8 dpi, with a significant decrease after 8 dpi, nadir after 12 dpi, and extremely slow growth after 16 dpi (Fig. 1B).

The survival analysis showed that only the mice in the 10^5^ PFU and 10^6^ PFU groups developing severe neurological signs died on 8 and 12 dpi. During the follow-up period, no mortality was detected in the other groups (Fig. 1C).

### Behavioral motor deficits induced by JEV.

**FIG 2:**
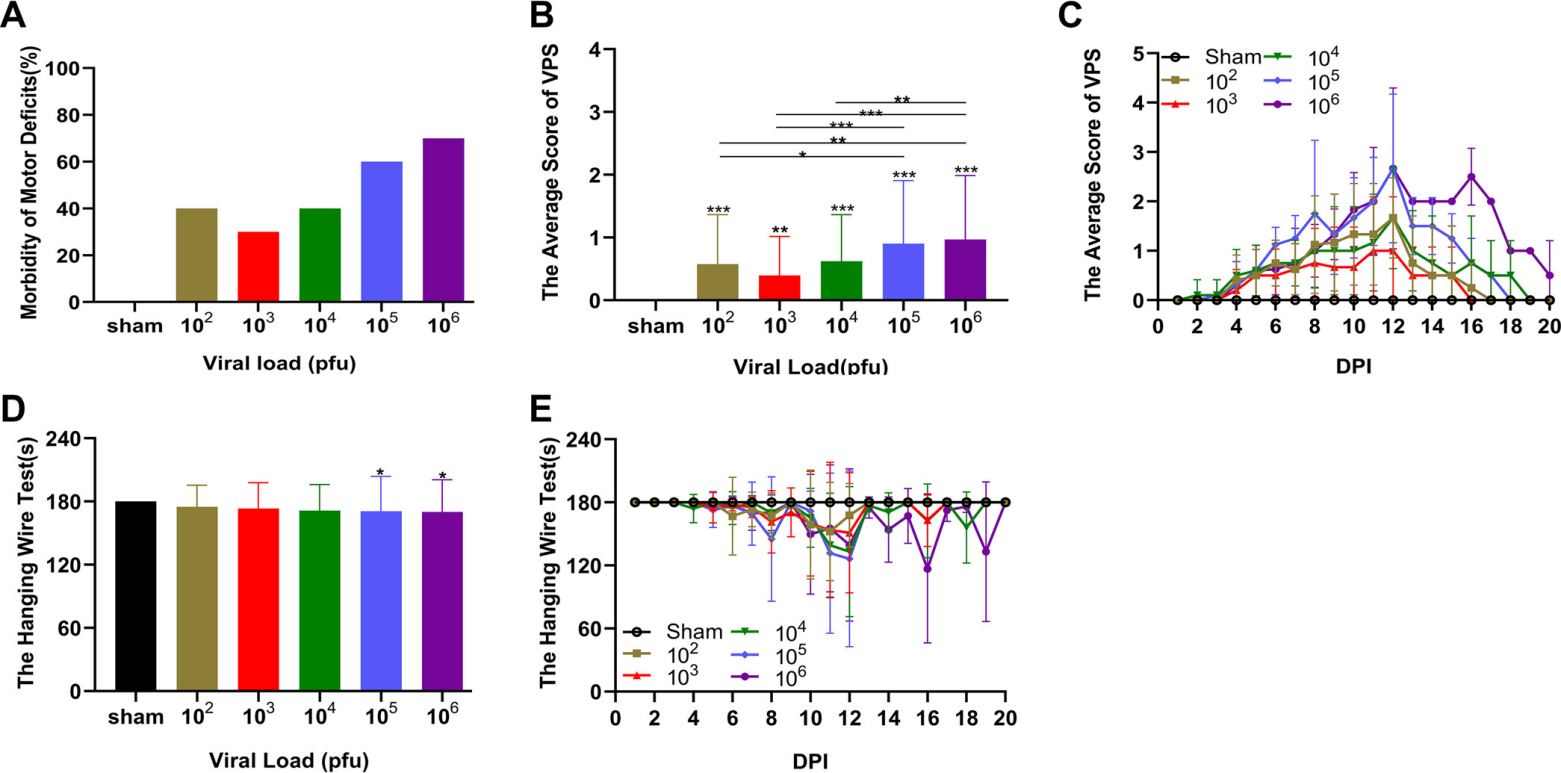
Motor deficits in JEV-inoculated mice. (A) The incidence rate of motor deficits of different groups (*n* = 10). (B) Mean VPS scores of different groups (*n* = 10). Differences among multiple groups were determined with one-way ANOVA followed with Bonferroni’s multiple comparison test. (C) Mean VPS scores were monitored 20 days after the virus challenge. (D) Mean hanging wire test scores of the different groups (*n* = 10). Kruskal-Wallis H test was applied for comparing multiple groups. (E) Mean hanging wire test scores were monitored for 20 days after the virus challenge. Data are expressed as means ± SD. *, *P* < 0.05; **, *P* < 0.01; ***, *P* < 0.001.

Only a subset of the mice inoculated with JEV presented motor deficits (incidence as follows: sham group, 0%; 10^2^ PFU group, 40%; 10^3^ PFU group, 30%; 10^4^ PFU group, 40%; 10^5^ PFU group, 60%; 10^6^ PFU group, 70%) (Fig. 2A). A similar phenomenon was observed in human JE. Conversely, the sham group did not present any joint rigidity or limb paralysis, as deduced from the viral paresis scale (VPS) score of 0. Virus-exposed mice displayed an increased incidence of tail dragging, missteps, stiffness, non-weight-bearing, and limb paralysis. The results of VPS revealed that the impairment of motor function in the 10^6^ PFU group was more severe than that in the 10^2^ PFU, 10^3^ PFU, and 10^4^ PFU groups (*P* = 0.0012, 0.0001, and 0.0075, respectively). No differences were observed between the 10^5^ PFU and 10^6^ PFU groups (Fig. 2B). The onset of marked motor function deficits detected by VPS ranged from 4 to 16 dpi. The initial motor deficits were observed on 4 to 5 dpi. The disease progressed rapidly and deteriorated persistently on 8 to 12 dpi. The motor function initiated a gradual recovery. However, the duration of the motor function deficits was significantly longer in the 10^6^ PFU and 10^5^ PFU groups compared to the other viral groups (Fig. 2C).

The hanging wire test was an objective method to observe the hind limb defects. Only the 10^6^ PFU (170.1 ± 30.6 s) and 10^5^ PFU (170.8 ± 33.1 s) groups showed statistically significant differences compared to the sham group (180.0 ± 0 s; *P* = 0.02 and 0.04, respectively), while no distinct difference was observed in the degree of motor function impairment among the viral groups (Fig. 2D). For the developmental process of the motor function deficits, the hanging wire test was closely parallel to the viral paresis scale test (Fig. 2E). In summary, escalating viral challenge dose did increase the severity of motor dysfunction. These results were in agreement with those obtained from survival studies.

### Determination of viral load in tissues from JEV-inoculated mice.

**FIG 3:**
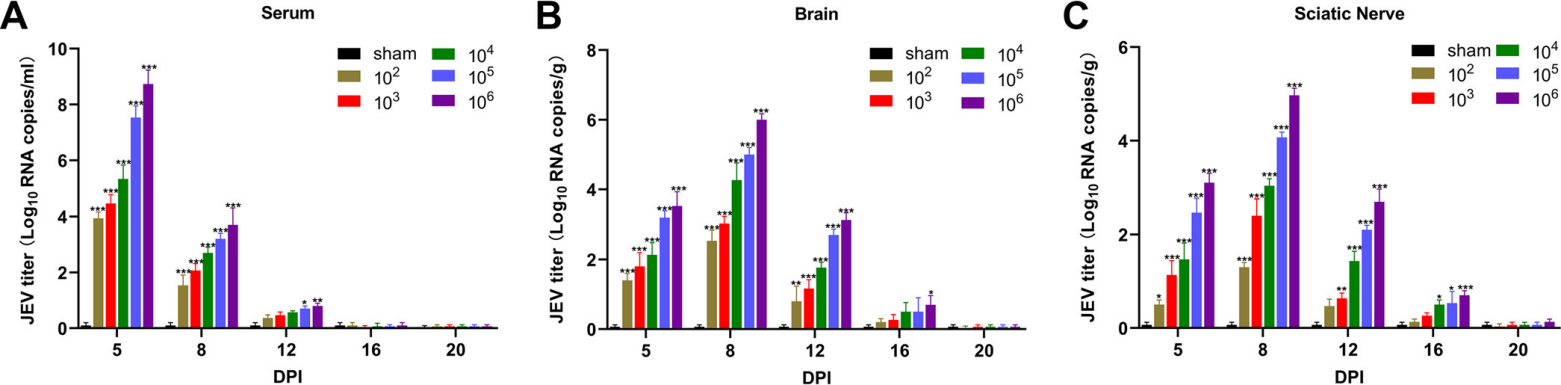
Viral RNAs in various tissues in the different groups of C57BL/6 mice after JEV infection during disease progression. (A) Serum; (B) brain; (C) sciatic nerve. The viral RNA titers of tissue samples obtained at five time points during infection were measured by RT-qPCR. Each bar represented the mean of three mice in the sham- and virus-infected groups. Two-way ANOVA with Tukey’s multiple comparison test was used (each viral group compared to sham). Asterisks indicated that values are statistically significant (*, *P* < 0.05; **, *P* < 0.01; ***, *P* < 0.001) compared to the results of sham mice.

To confirm whether the virus could infect the mice productively, reverse transcriptase quantitative PCR (RT-qPCR) was performed on mice. JEV RNAs were undertaken in the serum on 5, 8, and 12 dpi. The peak RNA titers were detected on 5 dpi, followed by a slow decline. The virus in the serum of the mice was eliminated at 16 dpi (Fig. 3A). Viral RNAs were also detected in the brain at 5 dpi, increased steadily, and reached the highest titers at 8 dpi, and finally it became virus-free at 20 dpi (Fig. 3B). In the sciatic nerves, increased JEV replication was observed on 5 dpi and remained elevated on 8 dpi; the viral copies declined from 12 dpi and became undetectable on 20 dpi (Fig. 3C). The average viral titers of the 10^5^ PFU and 10^6^ PFU groups were the highest in the serum (10^5^ PFU group, 7.5 log_10_ RNA copies/mL; 10^6^ PFU group, 8.7 log_10_ RNA copies/mL), brain (10^5^ PFU group, 5.0 log_10_ RNA copies/g; 10^6^ PFU group, 6.0 log_10_ RNA copies/g), and sciatic nerves (10^5^ PFU group, 4.1 log_10_ RNA copies/g; 10^6^ PFU group, 5.0 log_10_ RNA copies/g).

### Electrophysiological assessment of the sciatic nerve.

**FIG 4:**
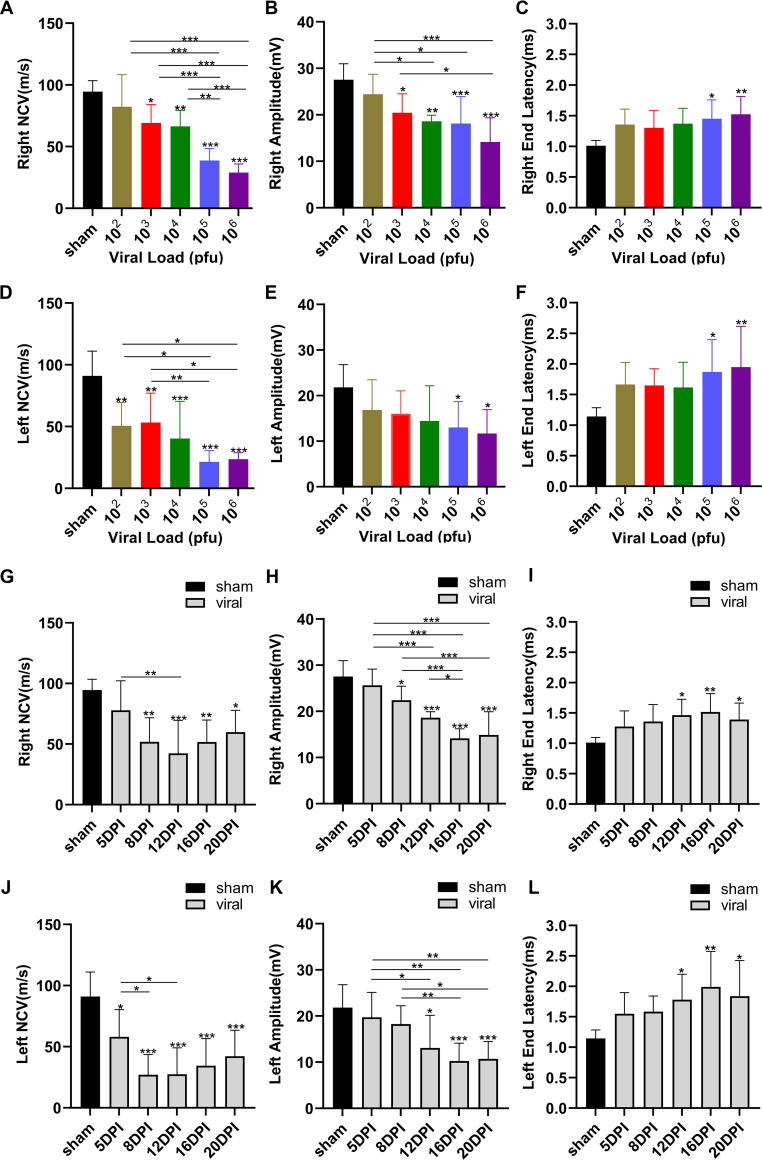
Sciatic nerve injury of JEV-inoculated mouse measured by electromyography. (A and D) Nerve conduction velocity in different groups. (B and E) Amplitudes in different groups. (C and F) End latency in different groups. (G and J) Nerve conduction velocity on 5, 8, 12, 16, and 20 dpi. (H and K) Amplitudes on 5, 8, 12, 16, and 20 dpi. (I and L) End latency on 5, 8, 12, 16, and 20 dpi. Data are presented as means ± SD. Comparisons among multiple groups were performed using one-way ANOVA followed by Tukey’s multiple comparison test. *, *P* < 0.05; **, *P* < 0.01; ***, *P* < 0.001.

In order to further assess the motor deficits in the mouse model after JEV inoculation, the left and right hindlimbs were examined by EMG. Nerve conduction velocity (NCV) was significantly slower in the viral groups compared to the sham group. Subsequently, pairwise comparisons were made between different viral groups. The results represented that NCV on the right hindlimb in the 10^5^ PFU and 10^6^ PFU groups showed significant differences compared to the other viral groups (Fig. 4A). On the left hindlimb, only 10^5^ PFU and 10^6^ PFU groups were different compared to the 10^2^ PFU and 10^3^ PFU groups (Fig. 4D). Moreover, NCV was significantly slower on 8, 12, 16, and 20 dpi, and the slowest conduction velocity was on 12 dpi (Fig. 4G and J). These studies indicated the presence of demyelinating peripheral neuropathy with reduced NCV.

On the right hindlimb, the amplitudes were decreased in JEV-inoculated mice except in the 10^2^ PFU group. The 10^2^ PFU group differed from the 10^4^ PFU, 10^5^ PFU, and 10^6^ PFU groups. In comparison, the 10^3^ PFU group showed differences from the 10^6^ PFU group (Fig. 4B). On the left hindlimb, the amplitudes declined in the 10^5^ PFU and 10^6^ PFU groups compared to the sham group. No differences were detected among the viral groups (Fig. 4E). An apparent decrease in the amplitudes on 12, 16, and 20 dpi (Fig. 4H and K). In addition, prolonged end latency was detected in 10^5^ PFU and 10^6^ PFU groups on the hindlimb (Fig. 4C and F). The most prolonged end-latency occurred on 12, 16, and 20 dpi (Fig. 4I and L). The decreased amplitudes and prolonged end latency revealed axonal-type motor neuropathy.

### Sciatic nerve injury detected by transmission electron microscopy analysis.

**FIG 5:**
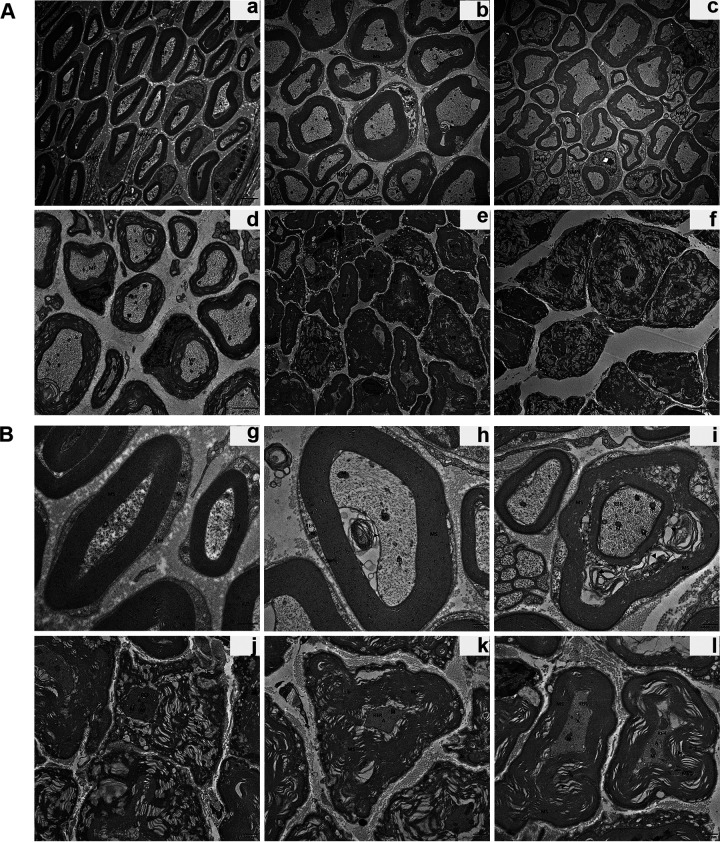
Ultrastructural abnormalities of mouse sciatic nerve after JEV infection detected by TEM. (A) Ultrathin cross sections of different groups (scale bar = 2 μm). (a) Sham group. (b) 10^2^ PFU group. (c) 10^3^ PFU group. (d) 10^4^ PFU group. (e) 10^5^ PFU group. (f) 10^6^ PFU group. (B) Ultrathin cross sections of the sciatic nerves at different time points (scale bar = 500 nm). (g) Sham group. (h) 5 dpi. (i) 8 dpi. (j) 12 dpi. (k) 16 dpi. (l) 20 dpi. MF, myelinated nerve; NMN, unmyelinated nerve; MS, myelin; A, axon; SC, Schwann cell; M, mitochondrial; RER, endoplasmic reticulum; ▴, demyelination.

In the sham group, the sciatic nerve was intact, the normal nerve fibers were arranged orderly, and the myelin sheath was thick and regular. Axons were surrounded by a myelin sheath and were not atrophic or swollen. Schwann cells and mitochondria were normal, while the sciatic nerve in the virus groups exhibited varying degrees of demyelination and axonal degeneration. Loosened myelin sheath and mild demyelination were observed in the 10^2^ PFU and 10^3^ PFU groups, and some axons showed mild atrophy and mutation. Next, we found mild edema of nerve fibers, slightly swollen mitochondria, abundant organelles, and structurally intact Schwann cells. In contrast to myelinated axons observed in the low-titer virus group, a majority of myelinated axons were remarkably reduced in the high-titer virus group (10^4^ PFU, 10^5^ PFU, and 10^6^ PFU). We also observed severe axonal atrophy, Wallerian degeneration, obvious demyelination, myelin lamellae disintegration, myelin dissolution, swollen mitochondria, dispersed and swollen endoplasmic reticulum, and distinctly swollen Schwann cells (Fig. 5A). Moreover, the ultrastructure examinations of axon and myelin at different time points revealed that demyelination was dominant in the early stage. As the process progressed, axonal atrophy and degeneration were evident, accompanied by severe demyelination; myelin regeneration and phagocytic clearance of myelin debris occurred during recovery (Fig. 5B).

### Sciatic nerve injury was associated with JEV infection.

**FIG 6:**
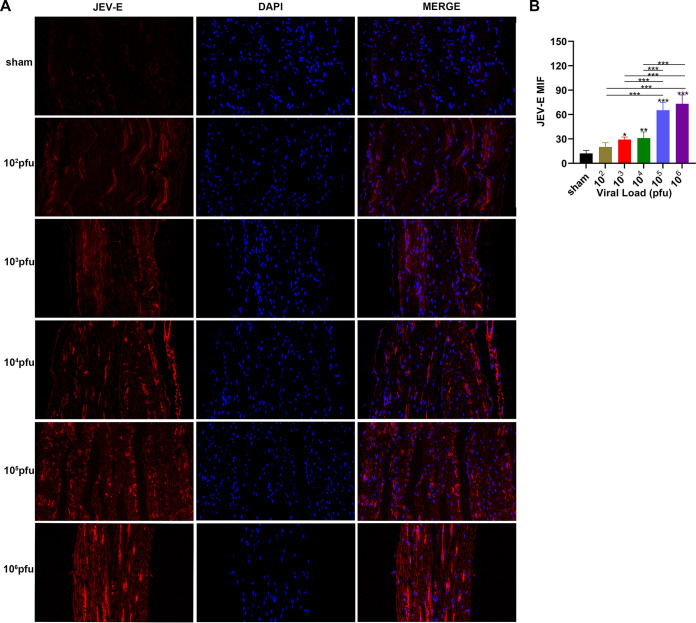
JEV attached to the mouse sciatic nerve. (A) Immunofluorescence staining of JEV-E protein (red) in different groups. DAPI was used to stain the cell nucleus (blue) (longitudinal section; scale bar = 20 μm). (B) Quantification of mean immunofluorescence intensity of JEV-E protein in different groups (*n* = 5). Data are presented as means ± SD. One-way ANOVA with Bonferroni’s multiple comparison test was performed to compare differences among multiple groups. *, *P* < 0.05; **, *P* < 0.01; ***, *P* < 0.001.

**FIG 7:**
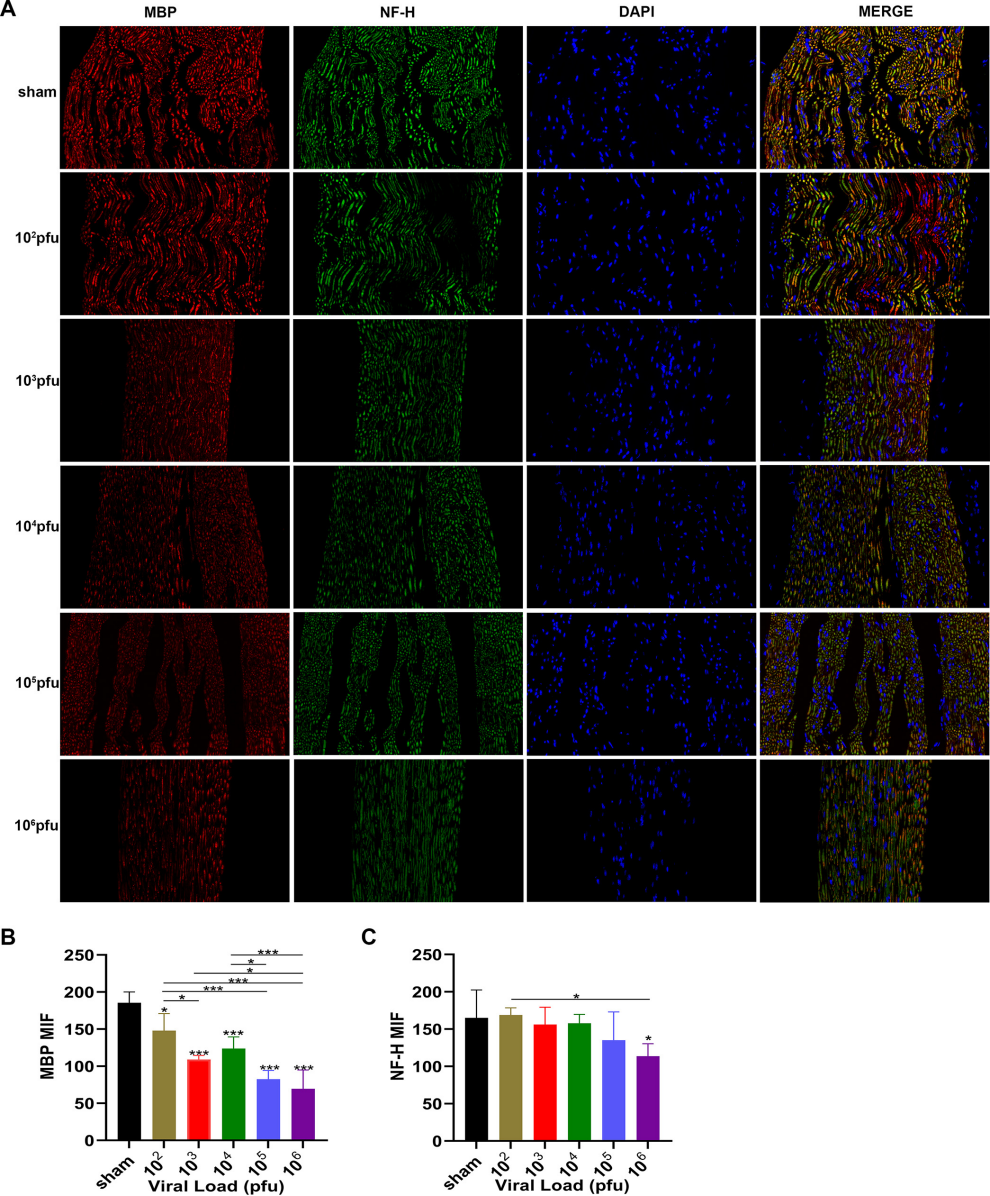
Axonal degeneration and demyelination appeared after JEV infection. (A) Immunofluorescence staining of MBP (red) and NF-H (green) in different groups. DAPI was used to stain the cell nucleus (blue) (longitudinal section; scale bar = 20 μm). (B) Quantification of mean immunofluorescence intensity of MBP in different groups (*n* = 3). (C) Quantification of mean immunofluorescence intensity of NF-H in different groups (*n* = 3). Data are presented as means ± SD. One-way ANOVA with Bonferroni’s multiple comparison test was performed to compare differences among multiple groups. *, *P* < 0.05; **, *P* < 0.01; ***, *P* < 0.001.

**FIG 8:**
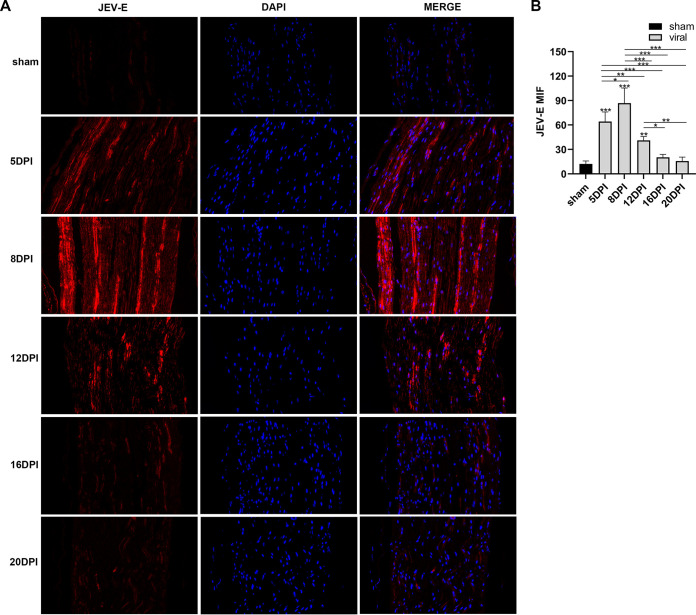
JEV-E protein expression in the sciatic nerve. (A) Immunofluorescence staining of JEV-E protein (red) at different time points. DAPI was used to stain the cell nucleus (blue) (longitudinal section; scale bar = 20 μm). (B) Quantification of mean immunofluorescence intensity of JEV-E protein at different time points (*n* = 3). Data are presented as means ± SD. One-way ANOVA with Bonferroni’s multiple comparison test was performed to compare differences among multiple groups. *, *P* < 0.05; **, *P* < 0.01; ***, *P* < 0.001.

**FIG 9:**
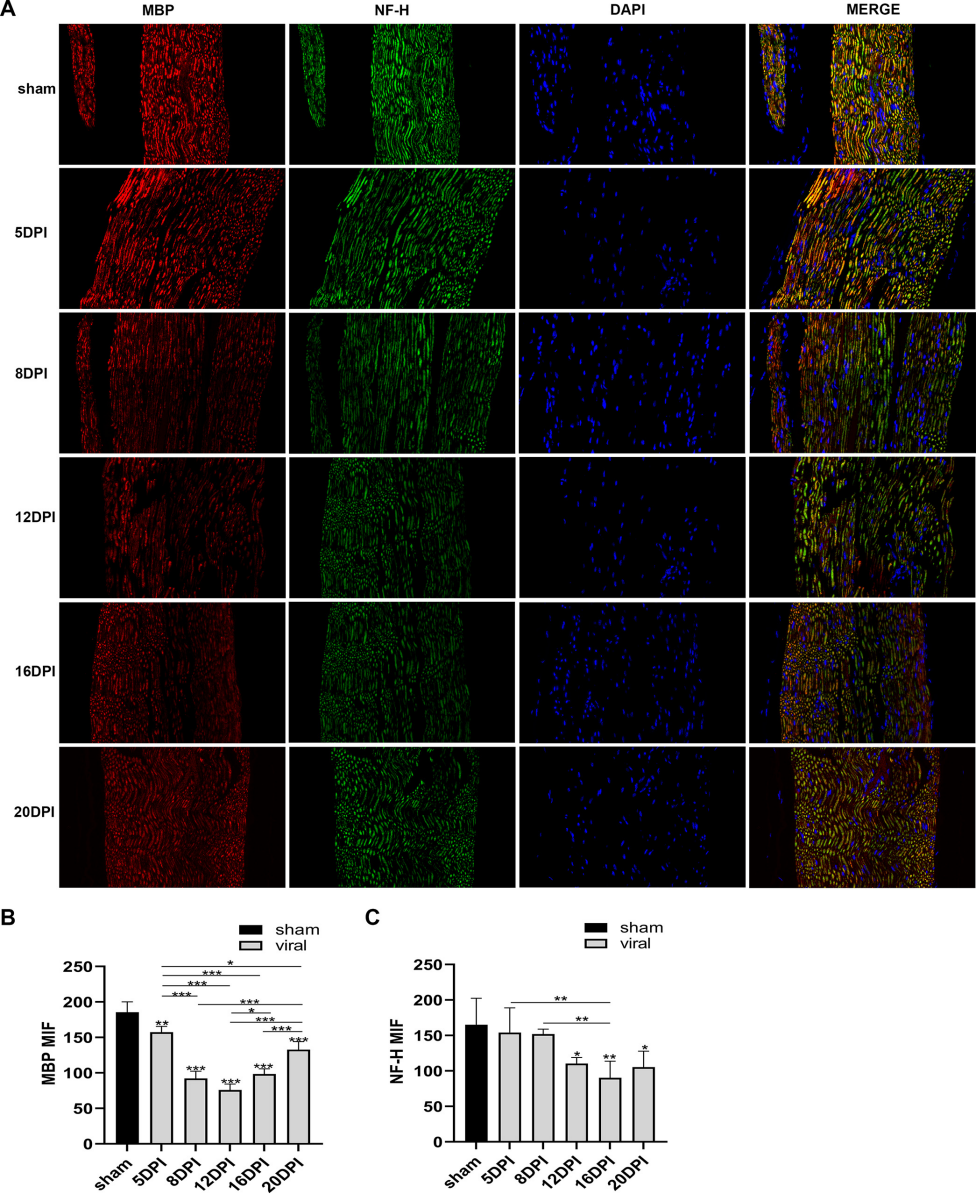
Axonal degeneration and demyelination appeared after JEV infection. (A) Immunofluorescence staining of MBP (red) and NF-H (green) at different time points. DAPI was used to stain the cell nucleus (blue) (longitudinal section; scale bar = 20 μm). (B) Quantification of mean immunofluorescence intensity of MBP at different time points (*n* = 3). (C) Quantification of mean immunofluorescence intensity of NF-H at different time points (*n* = 3). Data are presented as means ± SD. One-way ANOVA with Bonferroni’s multiple comparison test was used to compare differences among multiple groups. *, *P* < 0.05; **, *P* < 0.01; ***, *P* < 0.001.

We detected the expression levels of JEV-E protein, MBP (myelin sheath), and NF-H (axon) by immunofluorescence staining. The results indicated that the mean fluorescence intensity of E protein staining was increased markedly in the 10^5^ PFU and 10^6^ PFU groups compared to that in the sham group (Fig. 6A and B). The mean fluorescence intensity of MBP staining was decreased obviously in the virus group compared to that in the sham group. The 10^6^ PFU group had significant differences with the 10^2^ PFU, 10^3^ PFU, and 10^4^ PFU groups. The results of NF-H staining revealed significant differences between the 10^6^ PFU and sham groups. Other viral groups did not significantly differ from the sham group (Fig. 7A to C). JEV-E protein was abundant and broadly distributed in the sciatic nerve at 5 dpi, peaked at 8 dpi, the fluorescence intensity of E protein weakened from 8 to 16 dpi, and no JEV-E protein was detected at 20 dpi (Fig. 8A and B). The MBP expression was distinctly decreased on 8, 12, and 16 dpi, but NF-H staining was reduced after 12 dpi (Fig. 9A to C). Both viral RNA and E protein levels exhibited similar outcomes. In the early stages of JEV infection, the virus replicates in large quantities in the sciatic nerves; hence, we speculated that the axon and myelin injury of the peripheral nervous system is relevant to JEV infection.

### Histopathological changes and mRNA expression of inflammatory cytokines in the damaged sciatic nerve in mice.

**FIG 10:**
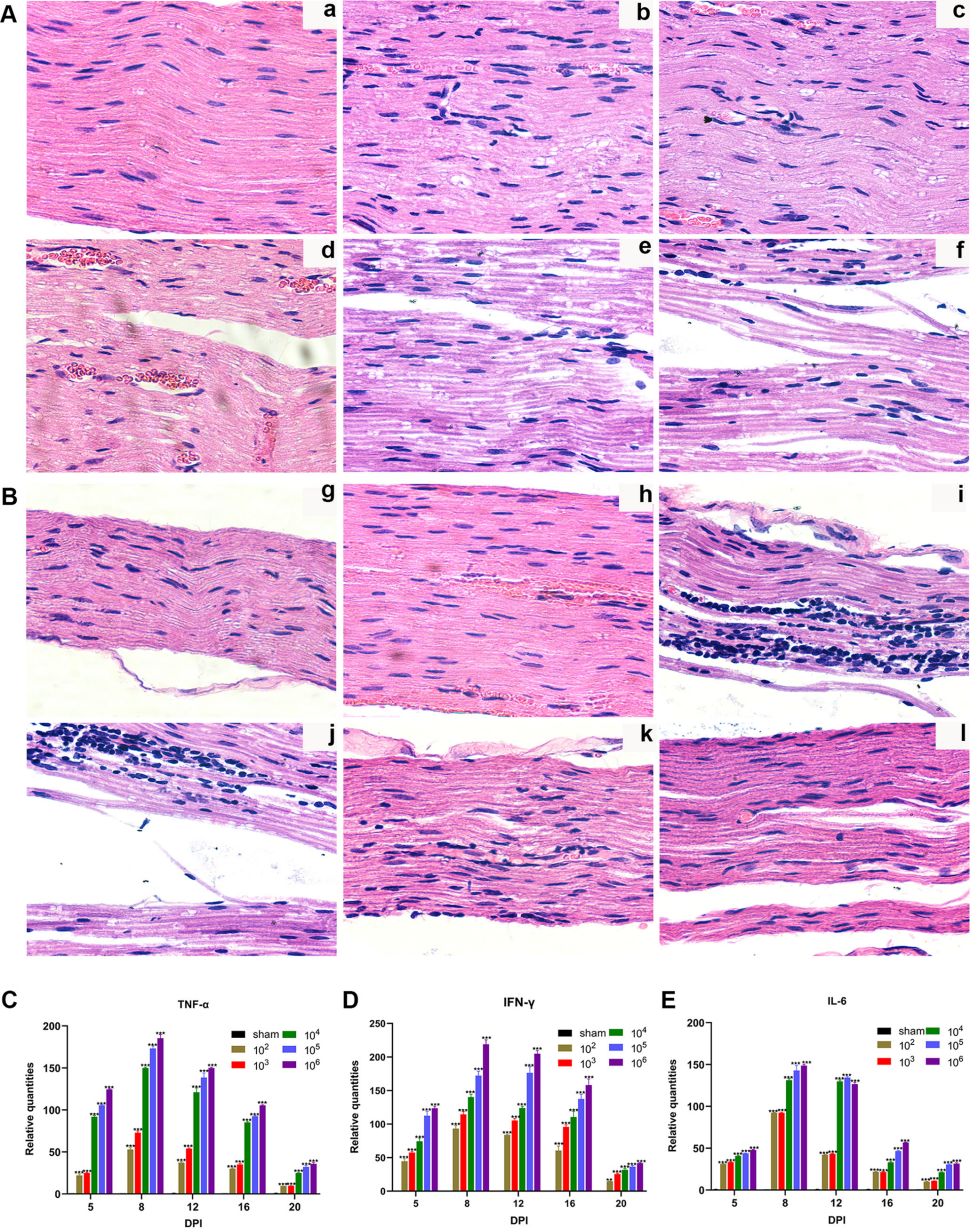
Histopathological changes and expression of inflammatory cytokines in mouse sciatic nerve. (A) Longitudinal section of the sciatic nerve in different groups (HE, 40×). (a) Sham group. (b) 10^2^ PFU group. (c) 10^3^ PFU group. (d) 10^4^ PFU group. (e) 10^5^ PFU group. (f) 10^6^ PFU group. (B) Longitudinal sections of the sciatic nerve at different time points (HE, 40×). (g) Sham group. (h) 5 dpi. (i) 8 dpi. (j) 12 dpi. (k) 16 dpi. (l) 20 dpi. (C) Relative mRNA expression levels of TNF-α. (D) Relative mRNA expression levels of IFN-γ. (E) Relative mRNA expression levels of IL-6. Data are presented as means ± SD. Two-way ANOVA with Bonferroni’s *post hoc* test was conducted (each viral group compared to sham). *, *P* < 0.05; **, *P* < 0.01; ***, *P* < 0.001.

The pathological results showed that myelinated nerve fibers were tightly arranged, and the shapes of axons were equal and regular without inflammatory cell infiltration in the sham group. No hyperemia or edema was observed. In 10^2^ PFU and 10^3^ PFU groups, a few nerve fibers started to swell, with a small number of inflammatory cells infiltrating, and the number of Schwann cells was slightly reduced. In the 10^4^ PFU, 10^5^ PFU, and 10^6^ PFU groups, the nerve fibers showed noticeable swelling, and the myelinated nerve fibers were irregular and loosely arranged. Schwann cells were visibly reduced. Conversely, inflammatory cell infiltration and apparent congestion in the epineurium were detected (Fig. 10A). At 5 dpi, the nerve fibers were morphologically intact and arranged neatly with visible swelling. Also, diffuse swelling of nerve fibers and infiltration of inflammatory cells were observed on 8, 12, and 16 dpi in addition to hemorrhage. At 20 dpi, the swelling of the nerve fibers gradually subsided, and the inflammatory cell infiltration declined (Fig. 10B).

Additionally, mRNA expression of the inflammatory cytokines was evaluated to determine the changes in the inflammatory response in the sciatic nerve. Tumor necrosis factor alpha (TNF-α), gamma interferon (IFN-γ), and interleukin-6 (IL-6) expressions were upregulated from 5 dpi, increased rapidly to 8 dpi, and decreased gradually to a low level. A significant difference was observed in the expression of TNF-α, IFN-γ, and IL-6 between sham-infected and virus-infected mice (Fig. 10C to E). Thus, a clear correlation was established between increasing viral load and expression of inflammatory factors.

### JEV infected mouse brain tissue and led to severe brain injury.

**FIG 11:**
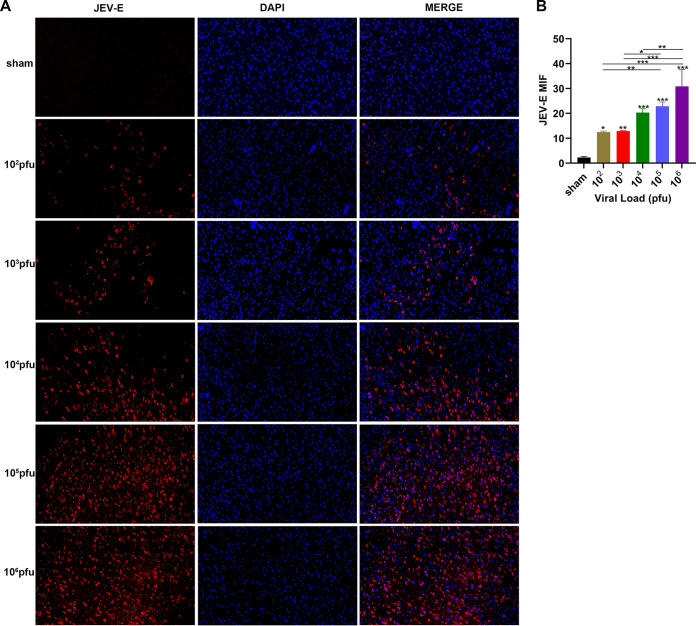
JEV attached to the mouse brain tissue. (A) Immunofluorescence staining of JEV-E protein (red) in different groups (coronal sections, scale bar = 50 μm). DAPI was used to stain the cell nucleus (blue). (B) Quantification of mean immunofluorescence intensity of JEV-E protein in different groups (*n* = 3). Data are presented as means ± SD. One-way ANOVA with Bonferroni’s multiple comparison test was performed to compare differences among multiple groups. *, *P* < 0.05; **, *P* < 0.01; ***, *P* < 0.001.

**FIG 12:**
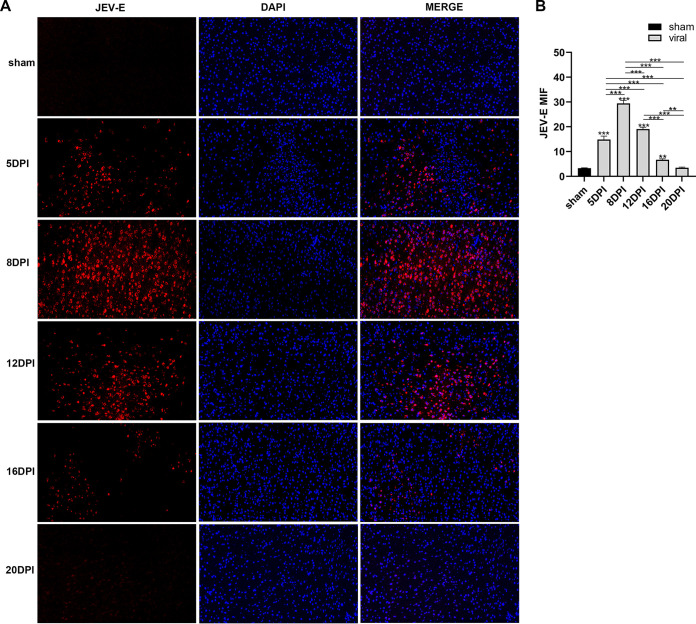
JEV-E protein expression in mouse brain tissue. (A) Immunofluorescent staining of JEV-E protein (red) at different times (coronal sections, scale bar = 50 μm). DAPI was used to stain the cell nucleus (blue). (B) Quantification of mean immunofluorescence intensity of JEV-E protein at different time points (*n* = 3). Data are presented as means ± SD. One-way ANOVA with Bonferroni’s multiple comparison test was used to compare differences among multiple groups. *, *P* < 0.05; **, *P* < 0.01; ***, *P* < 0.001.

JEV-E protein in the mouse brain was assessed by immunofluorescence. The current study showed a widespread distribution of JEV-E protein in the mouse brain in viral groups compared to that of the sham group. Increasing the viral titer elevated the expression level of JEV-E protein, and the 10^6^ PFU group had the highest amount of E protein (Fig. 11A and B). On 5 dpi, a significant increase was detected in the JEV-E protein levels, which peaked at 8 dpi, declined at 12 to 16 dpi, and was undetectable at 20 dpi (Fig. 12A and B).

**FIG 13:**
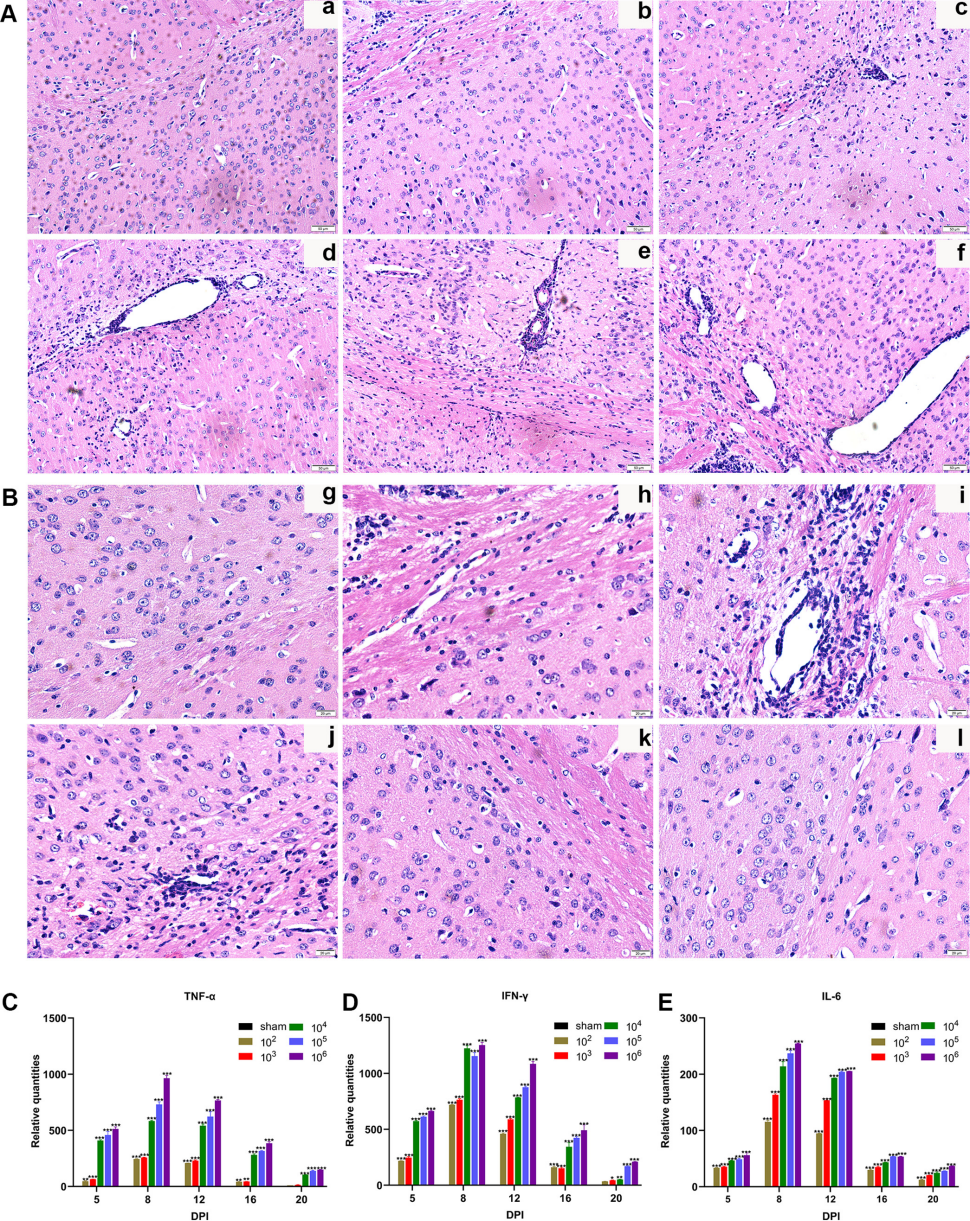
JEV infection caused neuronal cell death by activating the inflammatory response. (A) Histopathological changes in different groups (HE, scale bar = 50 μm). (a) Sham group. (b) 10^2^ PFU group. (c) 10^3^ PFU group. (d) 10^4^ PFU group. (e) 10^5^ PFU group. (f) 10^6^ PFU group. (B) Histopathological changes at different time points (HE, scale bar = 20 μm). (g) Sham group. (h) 5 dpi. (i) 8 dpi. (j) 12 dpi. (k) 16 dpi. (l) 20 dpi. Perivascular lymphocytic cuffing, neuronal necrosis, a glial nodule, and inflammatory cell infiltration were detected in the brain tissue. (C) Relative mRNA expression levels of TNF-α. (D) Relative mRNA expression levels of IFN-γ. (E) Relative mRNA expression levels of IL-6. Data were presented as means ± SD. Statistical difference was determined by two-way ANOVA with Tukey’s test (each viral group compared to sham). *, *P* < 0.05; **, *P* < 0.01; ***, *P* < 0.001.

Next, hematoxylin and eosin (HE) staining was performed, and the morphological structures of brain tissues were observed under a light microscope. The brain tissue structure was clearly visible in the sham-infected group with large round nuclei and abundant cytoplasm, while no tissue edema, hyperemia, hemorrhage, and inflammatory cell reaction was detected. Furthermore, increased edema, interstitial inflammation, and perivascular lymphohistiocytic cuffing were evident in the cortex, thalamus, and hippocampus of the JEV-inoculated mouse brain (Fig. 13A). On 5 dpi, a series of pathological phenomena were identified in the mouse brains, including infiltration of a few inflammatory cells, a small amount of perivascular lymphocytic cuffing, and occasional neuronal degeneration and necrosis. Conversely, massive inflammatory cell infiltration, the extensive proliferation of glial cells, broad neuronal degeneration, and necrosis were observed with sustained exacerbation of the lesions on 8 and 12 dpi. On 16 dpi, the congestion and edema of brain tissues and neuronal degeneration and necrosis were alleviated, and the lymphocyte infiltration was reduced gradually. On 20 dpi, the pathological changes in the mice brain tissue were barely seen, and the morphology recovered to normal (Fig. 13B).

Similar to the mRNA results of inflammatory cytokines of the sciatic nerve, elevated expression of TNF-α, IFN-γ, and IL-6 was observed in the brains of virus-infected mice at all of the time points. In the virus-infected group, the mRNA expression of TNF-α, IFN-γ, and IL-6 increased significantly from 5 to 8 dpi and downregulated slowly from 12 to 20 dpi. Also, inflammatory cytokines were upregulated with disease progression (Fig. 13C to E).

## DISCUSSION

Flaviviruses have strong neurotropic, neuroinvasive, and neurovirulent properties. They can invade the CNS and lead to severe and extensive encephalitis (2). It also has been widely reported that acute flaccid paralysis due to myelitis is directly related to dengue (32), Japanese encephalitis (33), Zika (34), and West Nile virus (35). GBS is the most frequent cause of acute flaccid paralysis worldwide and is often preceded by an infectious disease. Flavivirus infections are also responsible for a significant number of GBS patients, especially in highly endemic countries (36–38). However, GBS is rarely reported after JEV infection and is limited to case reports. During the outbreak of Japanese encephalitis in China in 2018, although 161 patients were confirmed with JEV infection through laboratory tests, and 47/161 JE patients were diagnosed with GBS by electromyography, JEV was isolated from only one case. This might be a new feature of JEV infection that was previously overlooked. In this study, we mainly focused on the JEV-induced peripheral nervous system injury.

A large number of the animal disease models are being used to elucidate the pathogenesis. Previously, a small-animal peripheral challenge model of JEV infection was developed using immunodeficient mice. As immune response plays a key role in the PNI induced by JEV, this model is not ideal for studying the pathogenic mechanism (39). Importantly, mice are naturally susceptible to JEV infection and develop clinical signs and pathological features similar to those in infected humans (40, 41). Age and route of inoculation are the key factors of susceptibility to flaviviruses in mice (15). Considering feasibility and noninvasion, we established a mouse PNI model by intraperitoneal injection of JEV. Our mice exhibited evident neurological signs, and mice infection data are consistent with previous findings (42). No distinctive difference was observed in the disease severity and pathological changes between the younger (4- to 6-weeks-old) and older (8- to 10-week-old) animals in the JEV-induced mouse model (43). Next, we observed that intraperitoneal JEV inoculation caused very low mortality but several effects on the nervous system in 6-week-old C57BL/6 mice. A robust and reproducible infection confirmed mice infection. Accumulating evidence from the mice data indicated that the injuries of the 10^5^ PFU and 10^6^ PFU groups were the most severe, leading us to conclude that the viral load might be positively correlated with disease severity, which was consistent with previous findings (21).

JEV-infected patients exhibited common symptoms and occasional neurological manifestations, such as GBS (44, 45). The case analysis results revealed that the GBS manifestations, such as hyporeflexia or areflexia, limb weakness or paresthesia, dysphagia, and respiratory muscle paralysis, are associated with JEV (46). Similar to the current findings, C57BL/6 mice were unable to maintain balance, often swayed, and fell during onset, indicating a connection with limb weakness and hyporeflexia. Some studies reported that subsets of the JEV-inoculated mice began to present mild and transient clinical signs at 3.5 dpi, while a majority of the animals exhibited typical neurological signs at 6 dpi that lasted up to 8 dpi (26). Our findings were similar to those of previous studies, but the typical neurological signs could persist much longer. The VPS and the hanging wire test illustrated that the mice inoculated with JEV had different degrees of motor function deficits, supporting the feasibility of C57BL/6 as an animal model for JEV-induced PNI.

Next, we described the clinical signs, severity, and course of the behavioral deficits in JEV-inoculated mice but could not determine whether such defects could be ascribed to encephalitis, myelitis, myositis, or peripheral neuropathy. In the current study, pathological changes are widely distributed throughout the brain. The typical pathological features of JE are nonsuppurative encephalitis, while neuron denaturation, cellular necrosis, reactive gliocyte hyperplasia, and perivascular cuffing are the most predominant manifestations. The neuropathological changes in the mouse brain are consistent with those in human autopsy samples (47–49). Furthermore, we focused on the dynamic pathological changes in the mouse brain tissue. At 5 dpi, JEV-E proteins were widely distributed in the mouse brain tissue with significantly increased viral RNA levels, confirming the strong neurotropism of this virus. The inflammatory changes are mild, with occasional perivascular cuffing, but without obvious neuron degenerative changes. As described in previous studies, from 8 to 12 dpi, proinflammatory cytokine expression was significantly increased with viral RNA levels and rising brain damage, characterized by inflammatory cell infiltrations, perivascular cuffing, and at the later stages, tissue necrosis. Interestingly, extensive viral replication in the brain tissue may directly cause neuronal cell death in the early stage. As the disease progresses, JEV may activate microglia, monocytes/macrophages, and neutrophils and induce neuronal cell death by inflammation-mediated cytotoxicity (2, 50, 51). The inflammatory reaction in the mouse brains represents the host immunological response to infected neurons. Thus, we could speculate that JEV infection results in severe brain damage.

The electrophysiological analysis of the sciatic nerve after JEV inoculation showed a marked reduction in amplitude, prolonged distal latencies, and reduced conduction velocities. GBS is an immune-mediated acute polyradiculoneuropathy involving the peripheral nervous system, causing progressive symmetric limb weakness with decreased or absent deep tendon reflexes, with ≤4 weeks of disease. The reduced motor and sensory nerve conduction velocities and the compound muscle action potential amplitudes are the typical electrophysiological features of GBS (52). The results of the EMG and the disease course induced by JEV are basically consistent with GBS. Also, the expressions of MBP and NF-H are weakened in the viral groups, indicating damage to the myelin sheath and axons in the sciatic nerve. Similarly, transmission electron microscopy (TEM) showed varying degrees of demyelination, axonal degeneration, and swollen mitochondria. The histopathological results supported these findings, wherein the sciatic nerve from the viral groups displays obvious edema of the myelinated nerve fiber. The predominant pathological features of GBS are demyelination of the nerve fibers, and in severe cases, axonal degeneration may be observed (53). The leading pathological changes in our model were consistent with the GBS phenomenon. Thus, we concluded that JEV infection results in varying degrees of injuries to the peripheral nervous system. Demyelination is the most common feature of peripheral nerve injury at the early stage, followed by axon degeneration and atrophy, and remyelination in the recovery stage. Myelin displays neurotrophic and neuroprotective effects, causing axonal injury to be the secondary response to myelination deficiency (54–56). Another study suggested that continuous communication between axons and myelin and the physiological and metabolic support of the myelin sheath are crucial during the development and maintenance of the axons. Remyelination also maintains axonal integrity, supports axonal metabolism, and aids neuronal transmission after inflammatory demyelination (57). Herein, we hypothesized that the recovery of nerve function is related to myelin regeneration. Mitochondrial injury also plays a critical role in driving axonal degeneration (58). A recent study suggested that inflammation is involved in the pathogenesis of peripheral nerve injury, which releases proinflammatory cytokines and attacks the myelin and axon (59). These results displayed a large amount of inflammatory cell infiltration and increased the inflammatory factors in the sciatic nerve. Hence, it could be speculated that JEV activates the inflammatory response by stimulating the inflammatory cytokines to cause peripheral nerve damage.

Previous studies reported that the earliest point at which JEV antigen is detected in the thalamus and medulla oblongata of the mouse is at 3 dpi. At 6 dpi, the virus is extensively distributed in the cerebral cortex, olfactory area, basal ganglia, thalamus, and brainstem areas. At 8 dpi, the viral antigen is widely distributed and highly concentrated in the whole brain, while no positive signal is observed in the cerebrum at 21 dpi (26). The current study showed that viral RNA levels and JEV-E protein are significantly increased in sciatic nerves and the brain. Moreover, the distribution time of JEV in the brain is consistent with that in the sciatic nerve. West Nile virus (WNV) can spread from axon terminals by long-distance retrograde axonal transport, infecting the CNS (60, 61). The spread of JEV may use neural pathways of the peripheral nervous system for brain invasion (62, 63). Due to the neurotropic nature of JEV, we speculated that JEV infects the sciatic nerve and directly causes PNI during peripheral nerve transport.

In conclusion, the combination of encephalitis and peripheral neuropathy might be ascribed to partial paralysis in our mouse model of JEV. Thus, it could be inferred that the mouse model of peripheral nerve injury induced by JEV was established successfully. Although viral RNA was elevated in the damaged sciatic nerve, the viral group inoculated with low titers had low RNA copies, i.e., <2 log_10_, and PNI may not be entirely attributed to the direct effects of the virus. The results of the proteomics analysis of JEV-induced PNI were combined with putative pathogenesis, including neuroinvasion, neuroinflammation, neuroimmune, metabolic dysfunction, neurodegeneration, mitochondrial dysfunction, and oxidative stress (39). Together, multifactorial pathogenic factors and mechanisms lead to the development and progression of PNI. Next, we will explore the pathogenesis of JEV-induced PNI to provide a basis for the prevention and treatment of the disease.

## MATERIALS AND METHODS

### Ethics statement.

This study was approved by the Institutional Animal Care and Use Committee of the Chinese Center for Disease Control and Prevention (China CDC approval numbers 20201110064 and 20210607040). Animal welfare was regularly monitored by veterinarians from the Service for Biotechnology and Animal Welfare. We followed all Animal Welfare Act regulations.

### Mouse.

Six-week-old male C57BL/6 mice, average weight 18 to 22 g, were purchased from Vital River (Beijing, China) and maintained in individually ventilated animal cages in the specific pathogen-free environment in the Animal Center of the China CDC. The animals were maintained at 20 ± 2°C, under a 12-h light/dark cycle and 50 to 70% humidity. Mice (*n* = 60) were randomly divided into six groups, five mice per cage. The general condition, including mouse hair, body weight, active status, survival status, and the development of clinical signs, were monitored daily during the experiment. Mice were sacrificed for sample collection on days 5, 8, 12, 16, and 20.

### Virus.

JEV GIb strain NX1889 was obtained from the Viral Diseases Prevention and Control Institute of China CDC. Whole-gene sequencing confirmed that the virus isolated from the cerebrospinal fluid (CSF) sample from a patient in Ningxia, China, 2018 was JEV GIb and named NX1889 (3). Five groups of mice (*n* = 10/group) were inoculated intraperitoneally with 10^6^ PFU, 10^5^ PFU, 10^4^ PFU, 10^3^ PFU, and 10^2^ PFU of the indicated viruses in a volume of 50 mL/mouse, respectively. The sham group was intraperitoneally injected at the same sites with the same dose of phosphate-buffered saline (PBS). All procedures were conducted at biosafety level 2 (BSL2) facilities with appropriate procedures.

### Behavioral motor function.

Viral paresis scales (VPS) (64) were used to assess paresis/paralysis of the hindlimb. Each mouse was placed on a platform and allowed to roam freely for 3 min. Four major classes were used to score as follows: trunk and tail control, miss-stepping, weight support, and joint movement (65). Severity was assessed daily and graded from 0 to 6 as follows: 0 = no clinical deficits—weight support, plantar stepping, erect tail; 1 = tail paralysis; 2 = hindlimb weakness or paresis—sideslip; 3 = hindlimb paresis—the paw could not touch the ground completely, significant limping; 4 = severe paresis—incomplete weight bearing, limb mostly dragging, still move forward; 5 = full hind limb paralysis—non-weight-bearing, joint rigidity; 6 = moribund or death (64, 65).

The hanging wire test evaluated neuromuscular strength and coordination. A 50-cm high-metal netting was placed above the table, covered with layers of soft towels to prevent injury in the event of falling. Mice were persistently suspended for 180 s, and the “falling” scores were recorded. If the animals hung on for 180 s, they were returned to their home cage, but two additional trials were conducted if the mice fell before the 180 s, with 2-mins between each test (a total of three trials).

### RNA extraction and qRT-PCR.

The brain and sciatic nerves were disrupted and homogenized by TissueLyser (30 Hz, 6 min) (Qiagen, Hilden, Germany). Serum was collected in the supernatant obtained by the centrifugation of blood samples. Viral RNAs were extracted using the Tianlong nucleic acid extraction kit on the nucleic acid automatic extraction instrument (Tianlong Biotechnology, Jiangsu, China). Gene expression was quantified by qPCR using the OneStep RT-PCR kit (Qiagen). The mRNA expression of inflammatory cytokines was assessed as follows: total RNA was extracted using an RNAsimple total RNA kit (Tiangen, Beijing, China). Reverse transcription was performed using PrimeScript RT reagent kit (TaKaRa, Japan). qPCR was conducted using TB Green Premix *Ex Taq* II (TaKaRa). The protocol was strictly in accordance with manufacturer’s instructions. The primer sequences were as follows: TNF-α, (forward) CGCTCTTCTGTCTACTGAACTTCGG, (reverse) GTGGTTTGTGAGTGTGAGGGTCTG; IFN-γ, (forward) CTGGAGGAACTGGCAAAAGGATGG, (reverse) GACGCTTATGTTGTTGCTGATGGC; IL-10, (forward) AGAGAAGCATGGCCCAGAAATCAAG, (reverse) CTTCACCTGCTCCACTGCCTTG.

### EMG.

After anesthetization, bilateral sciatic nerves were isolated from the mice. A thin needle wire placed in the tail coated with electrode gel served as the ground electrode. A stimulating needle electrode was placed at the sciatic exit position of the spinal nerve (proximal site), and a second stimulating needle electrode was placed at the sciatic notch (distal site) to obtain two distinct sites of stimulation along the nerve. The recording electrode was inserted into the ventral region of the gastrocnemius muscle. As the voltage increased gradually, the amplitude increased until it reached the peak. Motor amplitudes, conduction velocities, and end latencies were recorded by an NDI-096 electromyographic instrument (Haishen, Shanghai, China).

### Transmission electron microscopy.

Sciatic nerves were removed and immersed in 0.1 M phosphate buffer (PB), pH 7.4, containing 2.5% glutaraldehyde at 4°C overnight. After washing with 0.1 M PB thrice, the sciatic nerves were fixed for 2 h at room temperature with 1% osmic acid in 0.1 M PB, then washed with PB thrice for 15 min each. Subsequently, the samples were dehydrated in gradient alcohol and placed in 100% acetone for 20 min, 1:1 acetone/812 embedding agent for 3 to 4 h, 2:1 acetone/812 embedding medium infiltration overnight, and pure 812 embedding medium for 5 to 8 h; resin polymerization was at 60°C for 48 h. Ultrathin sections (60 to 80 nm) were sliced with ultramicrotome, followed by lead-uranium double-staining. The sections were dried at room temperature overnight, and the images were visualized under a TEM (ht7700; Hitachi, Japan).

### Immunofluorescence.

Mice were anesthetized with 10% chloral hydrate, followed by cardiac perfusion with 20 mL of PBS and then 50 mL of ice-cold 4% paraformaldehyde (PFA). Sciatic nerves and the brain were isolated and immersed in 4% PFA at 4°C overnight and then embedded in paraffin wax. Four-millimeter sections were cut and dewaxed in xylene twice for 15 min, followed by gradual rehydration with 100%, 85%, and 75% gradient alcohol and distilled water for 5 min. A microwave oven was used for antigen repair for 15 min on tissue sections placed in a repair box filled with EDTA antigen repair buffer (pH 8.0). After cooling to room temperature, the sections were washed three times, 5 min each time, with PBS (pH 7.4). The ring was filled with a spontaneous fluorescence quenching agent for 5 min and then washed with flowing water for 10 min. The sections were blocked with bovine serum albumin for 30 min and incubated with primary antibodies in PBS at 4°C overnight. The primary antibodies were as follows: rabbit anti-JEV-Envelope antibody (JEV-E) 1:500 (Genetex, Taiwan, China; GTX125867), rabbit anti-myelin basic protein monoclonal antibody (MBP) 1:800 (Abcam, Cambridge, UK; ab218011), and rabbit anti-neurofilament heavy polypeptide antibody (NF-H) 1:500 (Abcam, Cambridge, UK; ab207176). After three washes with PBS for 5 min each, the sections were incubated at room temperature for 50 min in the dark (CY3 goat anti-rabbit IgG [Servicebio, Wuhan, China; GB21303]; FITC goat anti-rabbit IgG [Servicebio; GB22303]). Finally, the slides were counterstained with DAPI (4′,6-diamidino-2-phenylindole) at room temperature for 10 min, sealed with an antifluorescence quencher, and visualized under a fluorescence microscope (Nikon eclipse C1, Japan); the images were captured by Nikon DS-U3.

### Hematoxylin and eosin staining.

The routine protocol for HE staining was followed. Briefly, after deparaffinization and rehydration, the nuclei of the sections were stained with hematoxylin for 5 min, diluted with 1% hydrochloric acid and alcohol for 10 s, and flushed again with fresh water for 15 min. After staining with eosin for 3 min, the slides were dehydrated in graded alcohol and cleared in xylene. Images were captured, and observations were analyzed using Olymbus.

### Statistical analysis.

Experiments were conducted in triplicate, and the results were presented as means ± standard deviation (SD). Data synthesis and analysis were performed using GraphPad Prism software version 9. The Shapiro-Wilk test was used to test the normality, and Levene’s test was applied to test the homogeneity of variance. Multiple comparisons were carried out by using one-way analysis of variance (ANOVA) followed by Bonferroni’s multiple comparison test or Tukey’s multiple comparison test. For comparisons of multiple factors, two-way ANOVA with Bonferroni’s *post hoc* test or Tukey’s multiple comparison test was used. The Kruskal-Wallis H test was used on data that were not normally distributed. *P* values of <0.05 were statistically significant.
