# Supplementary material for: Peripheral Nerve Injury Induced by Japanese Encephalitis Virus in C57BL/6 Mouse
Source: J Virol. 2023 Apr 18;97(5):e01658-22. doi: 10.1128/jvi.01658-22 (PMC10231255; doi:10.1128/jvi.01658-22)
Supplement: Supplemental file 1 — Supplemental material. Download jvi.01658-22-s0001.doc, DOC file, 4.0 MB [file jvi.01658-22-s0001.doc]

**
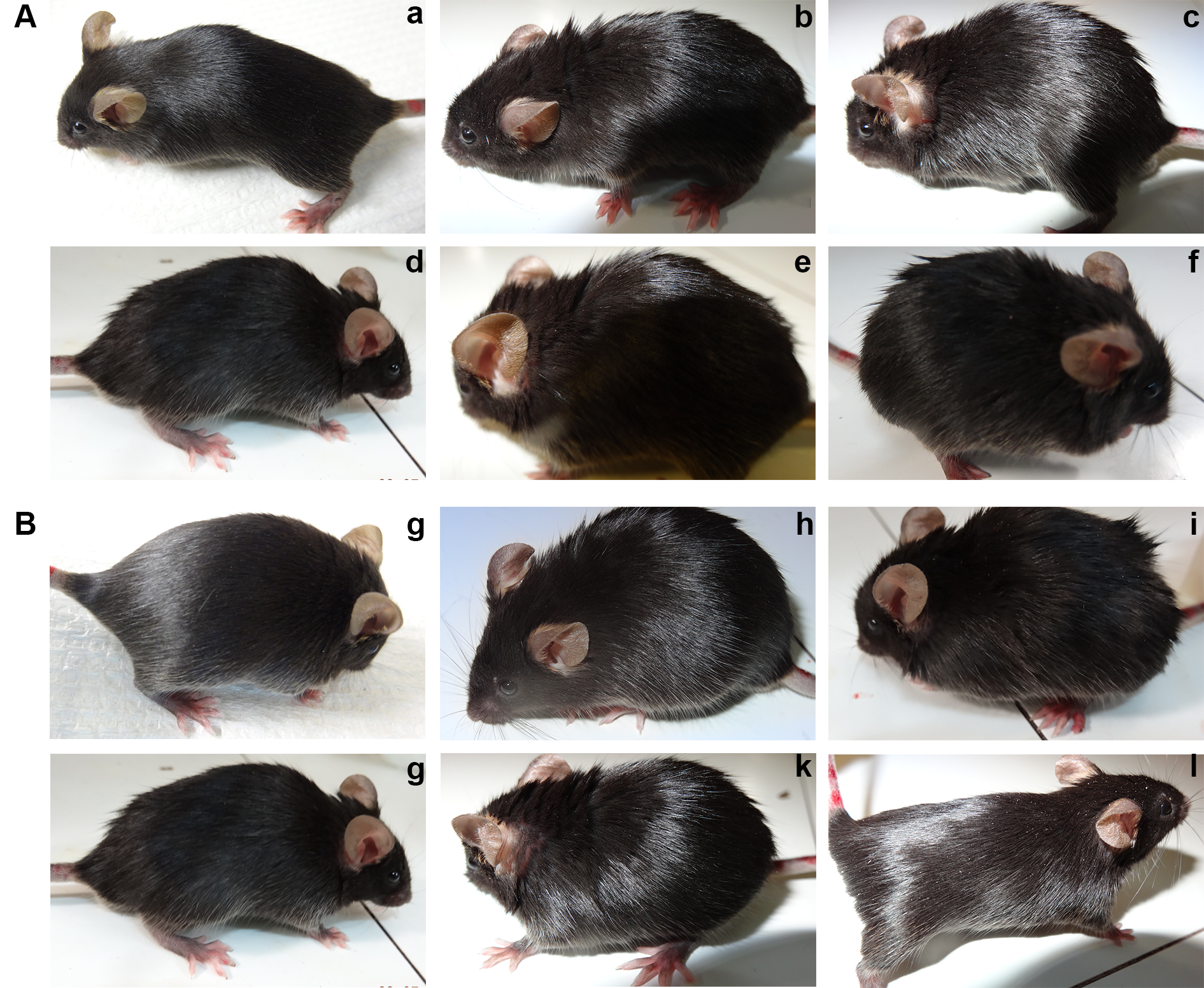
Supplement. 1** Changes in mouse hair after JEV infection. (A) Hair characteristics of the mice in different groups. (a) sham group; (b) 10^2^ pfu group; (c) 10^3^ pfu group; (d) 10^4^ pfu group; (e) 10^5^ pfu group; (f) 10^6^ pfu group. (B) Hair characteristics of the mice at different time point. (g) sham group; (h) 5 dpi; (i) 8 dpi; (j) 12 dpi; (k) 16 dpi; (l) 20 dpi.
